# Goal representation in the infant brain^☆^

**DOI:** 10.1016/j.neuroimage.2013.08.043

**Published:** 2014-01-15

**Authors:** Victoria Southgate, Katarina Begus, Sarah Lloyd-Fox, Valentina di Gangi, Antonia Hamilton

**Affiliations:** a Centre for Brain and Cognitive Development, Birkbeck College, Malet Street, London, UK; b Department of Developmental Psychology and Socialization, University of Padova, via Venezia 8, 35131 Padova, Italy; c School of Psychology, University of Nottingham, University Park, Nottingham, UK

**Keywords:** Functional near infrared spectroscopy, Goal attribution, Infants, Repetition suppression, Action understanding

## Abstract

It is well established that, from an early age, human infants interpret the movements of others as actions directed towards goals. However, the cognitive and neural mechanisms which underlie this ability are hotly debated. The current study was designed to identify brain regions involved in the representation of others' goals early in development. Studies with adults have demonstrated that the anterior intraparietal sulcus (aIPS) exhibits repetition suppression for repeated goals and a release from suppression for new goals, implicating this specific region in goal representation in adults. In the current study, we used a modified paired repetition suppression design with 9-month-old infants to identify which cortical regions are suppressed when the infant observes a repeated goal versus a new goal. We find a strikingly similar response pattern and location of activity as had been reported in adults; the only brain region displaying significant repetition suppression for repeated goals and a release from suppression for new goals was the left anterior parietal region. Not only does our data suggest that the left anterior parietal region is specialized for representing the goals of others' actions from early in life, this demonstration presents an opportunity to use this method and design to elucidate the debate over the mechanisms and cues which contribute to early action understanding.

## Highlights

• This study explored the brain bases of infant goal understanding
• We used fNIRS to identify the brain regions involved in goal representation
• Infants recruit the left parietal cortex when observing goal-directed actions
• Our findings enable future studies of mechanisms of early action understanding

## Introduction

From an early age, human infants interpret others' movements in terms of the goal towards which the movement is directed. Understanding the mechanisms that support action interpretation, and the development of the underlying brain systems, is important in the study of basic mechanisms of social interaction.

Previous studies of goal understanding in infants commonly measure the infant's looking responses. In one such paradigm, infants are repeatedly shown an agent acting upon one of two objects. After infants have seen this repeated action, the objects switch location, and the infant is presented with the agent acting again on the previously chosen object or acting on the previously un-chosen object. Infants from as early as three months of age respond with longer looking towards the event in which the agent acts on the previously un-chosen object (Luo, 2011; Sommerville et al., 2005), suggesting that they had encoded the prior events as movements directed towards a specific object (Woodward, 1998). In a different paradigm, infants repeatedly observe an agent acting towards an object in an efficient manner as dictated by the environment (e.g. reaching over an obstacle to contact an object). In subsequent events, the obstacle is removed and a direct reach becomes the most efficient means to achieving the same goal. In accord with this expectation, infants from at least six months of age respond with increased looking when the agent continues to perform a detour action when it is no longer necessary (Csibra, 2008; Kamewari et al., 2005; Southgate et al., 2008). This suggests that infants interpreted the previous action as directed towards the goal object and expected the agent to continue to pursue the same goal by the most efficient means (Gergely et al., 1995).

Recently, there has been much debate over what cues and mechanisms support early goal representation (Biro and Leslie, 2007; Hernik and Southgate, 2012; Kuhlmeier and Robson, 2012; Luo and Choi, 2012). Some studies have suggested that it is the infants' own experience with an action that provides them with a concept of an action as directed towards a goal (Hernik and Southgate, 2012; Woodward, 2009). Support for this position comes from studies showing that infants more readily attribute goals to actions that are part of their own motor repertoire (e.g. reaching actions) than actions which are novel (e.g. the approaching actions of a mechanical claw or a hand approaching an object in an unusual way) (Cannon and Woodward, 2012; Kanakogi and Itakura, 2011; Luo, 2011; Woodward, 1998). However, there is also substantial evidence that young infants can represent the goals of actions that are beyond their own motor experience. For example, infants represent the goals of actions performed by animated shapes (Csibra, 2008; Hernik and Southgate, 2012; Luo and Baillargeon, 2005), mechanical claws or rods (Biro and Leslie, 2007; Southgate and Begus, in press) and hands performing actions in unusual ways (Király et al., 2003; Southgate et al., 2008), none of which they could have first person experience on which to draw. These studies suggest that early goal representation may be more dependent on the availability of certain cues than prior experience with that action.

Which cues might be important for representing an action as goal-directed, and whether some cues have supremacy over others, is unclear. For example, it is often assumed that repetition of action on the same object is required for goal attribution (Luo and Beck, 2009; Premack and Premack, 1994) but other studies have demonstrated goal attribution in the absence of repeated action (Southgate and Csibra, 2009) and repeated action on a solitary object does not appear to result in goal attribution (Luo and Baillargeon, 2005, 2007). An additional or alternative basis for goal attribution may be the presence of an action that is selective; an action that is directed towards one object in the presence of another object seems to generate an interpretation that the action is goal-directed (Biro et al., 2011; Hernik and Southgate, 2012). As mentioned earlier, numerous studies have confirmed that infants appear to exploit cues to action efficiency for goal representation (Gergely et al., 1995), and some have proposed that efficiency may take precedence over cues to selectivity because infants apparently fail to represent an inefficient action directed towards one of two objects as a goal-directed action (Verschoor and Biro, 2011). However, it is nevertheless proposed that use of these different cues results in a unitary concept of goal, even in infancy (Biro et al., 2011). Finally, in the absence of alternative measures of goal representation, infants' failure to demonstrate the typical pattern of looking (e.g. equivalent looking towards actions directed to previously chosen vs. previously un-chosen objects) has become the litmus test for goal attribution, and such a reliance on one measure may be failing to provide an accurate picture of the underlying mechanisms (Kuhlmeier and Robson, 2012).

One way to elucidate these issues is to ask whether the same brain regions are recruited during the processing of events containing different cues, that ostensibly lead to the representation of a goal. Research in adults using fMRI has highlighted the inferior frontoparietal cortex as being involved in goal representation (Hamilton, 2006; Hamilton and Grafton, 2007a; Ramsey and Hamilton, 2010). Functional near infrared spectroscopy (fNIRS) can record activity of the equivalent brain regions in typically-developing infants whilst they observe goal-directed actions, providing the opportunity to interrogate the mechanisms underlying early goal attribution without requiring overt responses from the infant. The current study is a first step towards this aim.

Here, we investigate which cortical regions of the infant brain are involved in the processing of a simple goal-directed event. To this end, we used a repetition suppression (RS) design, similar to that used with adults, and which has previously identified regions of the cortex involved in goal representation (Hamilton, 2006; Hamilton and Grafton, 2007b). RS in response to the repeated presentation of a particular aspect of a stimulus, and a release from suppression when that aspect of the stimulus is changed, indicates that a particular brain region is sensitive to that property of the stimulus (Grill-Spector et al., 2006). Thus, in adults, the anterior intraparietal sulcus (aIPS) exhibits RS when the immediate goal of an action is repeated, but a release from suppression when the goal changes, strongly suggesting that the aIPS is involved in representing the goal of an action. Whilst a traditional blocked RS design has previously been employed in infants using fNIRS (Kobayashi et al., 2011), in the current study we used a paired RS design in which activation in response to individual test events is measured following a directly preceding establishing event (Kilner et al., 2009). Based on the fact that neural suppression in adults is clearly seen on a single repeated trial (Hamilton and Grafton, 2007b), and the need to obtain sufficient data from two conditions (Repeated Goal and New Goal) containing a lengthy dynamic event, a paradigm which measures activation on single test events that directly follow an establishing event provided the best design to localise goal representation in the infant brain.

Infants were presented with animations in which a red triangle detours around a barrier to collect one of two shapes (a blue square or a green triangle). In this way, the event contained several cues that are thought to enable infants to interpret an event as goal-directed (efficient action and selective outcome). Similar animations have previously been shown to be interpreted by 9-month-olds as goal-directed events (Hernik and Southgate, 2012), and to elicit activation in the anterior parietal cortex in adults (Ramsey and Hamilton, 2010). Based on the existing studies with adults, we hypothesized that infants would show greater activation in the left parietal cortex when viewing actions directed towards novel goals compared to actions directed towards repeated goals. This result would establish the validity and feasibility of FNIRS for exploring the mechanisms underlying the development of goal understanding in infants.

## Material and methods

### Participants

The final sample consisted of 18 9-month-old infants (11 males; mean age = 277 days, range = 263–297 days). An additional 22 infants were excluded due to fussiness (did not complete a minimum of 6 trials (13 infants)), positioning of the fNIRS headgear (poor placement/very large or small head (5 infants)), or due to excessive movement artefacts and/or inattention, which resulted in more than 30% of the contributed data being excluded (4 infants).

### Stimuli and design

**Fig. 1:**
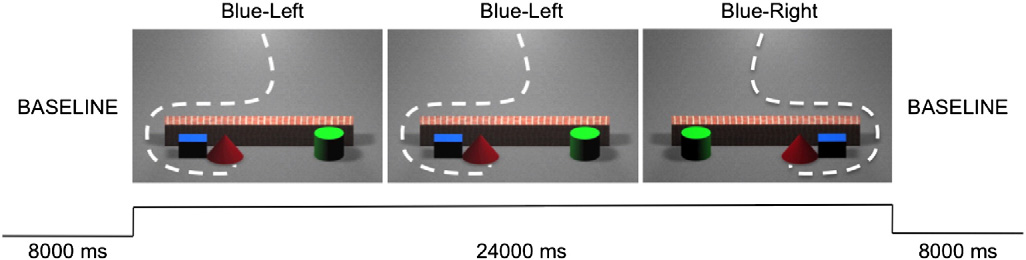
Experimental design depicting a Repeated Goal trial. Each trial consisted of three stimuli presented one after the other, interleaved with an 8 s baseline. The first two stimuli depicted the goal-establishing event (e.g. red cone approaching blue cube) and third served as the test trial in which either the goal remained the same (e.g. red cone approaches blue cube again) or changed (e.g. red cone approaches green cylinder). Side of target object was randomized.

Animations were created with Maxon Cinema 4D and presented on a 102 by 58 cm plasma screen with MATLAB. Each animation showed a red cone detouring around a barrier towards either a blue cube or a green cylinder (see Fig. 1). The red cone then ‘collected’ its target and returned to its starting position. Each animation lasted 7.5 s and animations were separated by a 0.5 second gap, giving a total trial duration of 24 s. Each trial was interleaved with an 8 second baseline in which infants saw changing images of houses, outdoor scenes, animals and faces.

The animations were presented to infants in a modified paired repetition suppression design (Kilner et al., 2009) in which each trial was composed of a set of three animations. The first two animations (Goal-Establishing event) showed the red cone moving towards one target object (either blue cube or green cylinder). The third animation showed either the red cone moving towards the same target (Repeated Goal event) or the red cone moving towards the other target (new goal event). For example, if the red cone approached the green cylinder in the first two events of the triplet, it would either continue to approach the green cylinder in the third event (Repeated Goal trial) or would approach the blue cube in the third event (new goal trial). We included two repetitions of the goal-establishing event to maximize the chance that infants identified the goal of the red cone by the time they were presented with the third event of the triplet. This design also meant that if infants did not attend during one of these goal-establishing events, but viewed the other one and the test trial, the data from the test trial could still be used. To isolate activation that was the result of a goal change rather than a path change, we counterbalanced the path that the red cone took towards its target (the target would either be located to the left or the right of the barrier) such that on some trials the path to the new goal would remain the same as that previously taken (the red cone which had previously approached the blue cube on the left would now approach the green cylinder on the left), or it would change (the red cone which had previously approached the blue cube on the left would now approach the green cylinder on the right). Thus, we had 16 different trials which were categorized in to 4 types: New Goal – New Trajectory (nGnT), Repeated Goal – New Trajectory (rGnT), New Goal-Repeated Trajectory (ngrt) and Repeated Goal-Repeated Trajectory (rGrT). Trials were presented in a pseudo-randomized order with a stipulation that, within every four trials, each trial type would be presented. As previous fNIRS studies have excluded infants with less than 3 trials per condition (Lloyd-Fox et al., 2011), our pseudo-randomization additionally stipulated that, within the first 6 trials, infants would be presented with equal numbers of repeated goal and new goal trials. This maximized our chances of obtaining sufficient data for analysis given the length of our trials.

### Procedure

#### fNIRS data acquisition

**Fig. 2:**
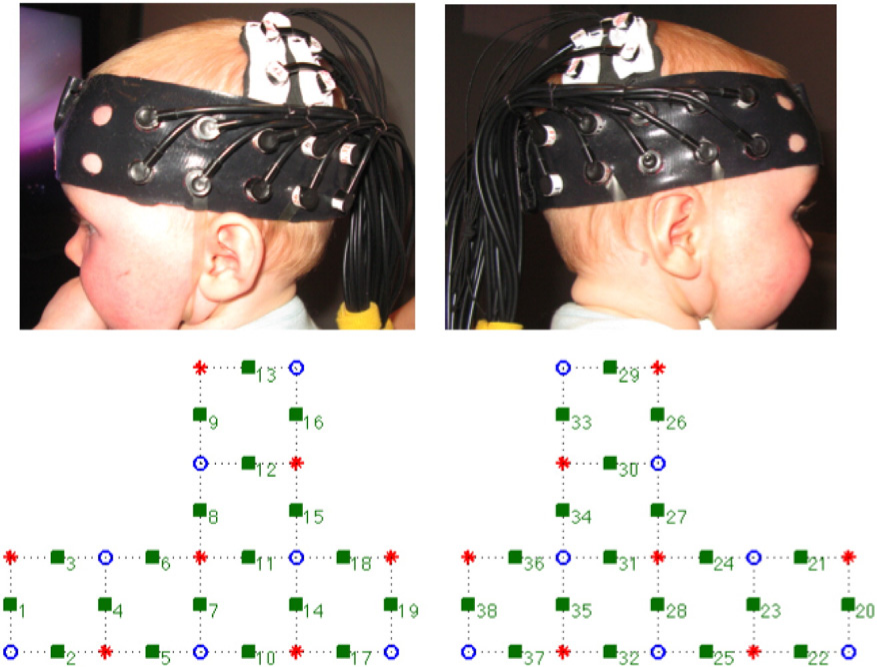
fNIRS headgear and channel layout. Top images show photo of left and right source/detector arrays as placed on infant head. Bottom images show location of sources (red stars), detectors (blue circles) and resulting channels (green squares).

To measure Hb concentration changes in the infant brain, we employed FNIRS (University College London topography system NTS; (Everdell et al., 2005)), using two continuous wavelengths of source light at 770 and 850 nm. Infants wore a custom-built headgear, consisting of two source-detector arrays (left and right hemisphere, see Fig. 2), containing a total of 38 channels, with source-detector separations at 2.5 cm. On the basis of an understanding of light transport and given that the cortex is approximately 0.75 cm from the skin surface in this age group (measure taken from structural MRIs, (Salamon et al., 1990)) the 2.5 cm channel separations used in the current study were predicted to penetrate up to a depth of approximately 1.25 cm from the skin surface, potentially allowing measurement of both the gyri and parts of the sulci near the surface of the cortex (Lloyd-Fox et al., 2010).

Before the infants began the study, head measurements (circumference; the distance between glabella, ears, and inion; distance between ears measured over the top of the head) were taken to align the headgear with the 10–20 coordinates (Jasper, 1958). With the use of age-appropriate infant structural MRIs, anatomical scalp landmarks, and the 10–20 system, we can approximate the location of underlying cortical regions for the infants, and draw comparisons of general regional activation with findings in adults. Measurements from the final sample of infants showed that the distance from the glabella to the ear (T3/T4) ranged from 11 to 12.5 cm (*M* = 11.8 cm, *SD* = 1.25 cm), and the distance between ears as measured over the top of the head ranged from 11.5 to 13.5 cm (*M* = 12.5 cm, *SD* = 1.15). The distance from the midpoint of the headband over the forehead (the glabella) to the channels above the ears (Channel 5 left hemisphere and Channel 25 right hemisphere) is fixed and aligned approximately with T3 and T4 of the 10–20 system on an average 9-month-old infant head (45 cm circumference; unpublished observation from the 100 + infants of this age range for which we have these measurements). This allowed the more dorsal channels (8, 9, 12, 13 (left) and 26, 27, 29, 30 (right)) to be positioned primarily over the supramarginal gyrus, the angular gyrus and the inferior parietal sulcus of the parietal lobe (the locations of the fNIRS channels on the scalp surface were co-registered with the closest underlying cortical areas on a nine-month-old infant MRI 3 T template using an MNI stereotaxic atlas from John Richards, personal communication; for general method see Richards, 2013).

#### Experimental procedure

Once the fNIRS headgear was fitted, infants were seated on their parents lap, approximately 140 cm from the screen. Infants watched the trial sequence whilst fNIRS data and video footage of the infant was recorded. Trials continued until the infant became inattentive or fussy. To maintain interest, 8 different sounds were played during the presentation of stimuli, in random order. During baseline, a sound was played at the beginning of each new image displayed (1 sound every 2 s) and during trials a sound was played at the beginning of each video and at the point where the animated shape made contact with its target. Infants received between 4 and 8 Repeated Goal trials (median = 6) and between 4 and 8 New Goal trials (median = 6). Infant looking towards the screen was analyzed off-line for attentiveness. Time points where the infant looked away from the screen were entered in to the analysis (see data processing and analysis below). Entire trials were excluded if the infant did not attend for 50% of at least one of the goal-establishing events and/or 50% of the test event. Trial exclusion resulted in included infants contributing between 3 and 8 Old Goal trials (median = 4) and between 4 and 7 New Goal trials (median = 5). As with previous studies (e.g. Lloyd-Fox et al., 2009), a minimum of 3 valid trials in each of the two conditions (Repeated Goal and New Goal) was required to include an infant in the final sample.

### Data processing and analysis

Data analysis was conducted using a combination of custom Matlab scripts and the SPM-NIRS toolbox (Ye et al., 2008). We took several steps to remove artefacts from the data. First, channels were excluded from the data if the coefficient of variation (std/mean) for all the data collected on that channel was over 10% (Lloyd-Fox et al., 2009). Any remaining channels that were continuously noisy were excluded based on visual inspection. Then, time periods affected by movement artefact were identified by (1) subtracting the mean signal from each channel (2) taking the absolute value of the signal in each channel (3) averaging the signal across time points. This gave a measure of the global signal strength over all channels, allowing us to identify movement artefact as spikes in the global signal. An artefact threshold was set for each infant by visual inspection of all the good data channels and the global signal; the threshold was set to exclude time points contaminated with clear movement artefact. Movement-induced artefact removal based on visual inspection has also been used in several other infant fNIRS studies (Minagawa-Kawai et al., 2011; Taga et al., 2003). The threshold for each infant was constant over the whole time course of the study and was set blind to the experimental condition. Data points in the time periods marked as ‘over threshold’ were then set to zero, effectively removing them from the analysis.

**Fig. 3:**
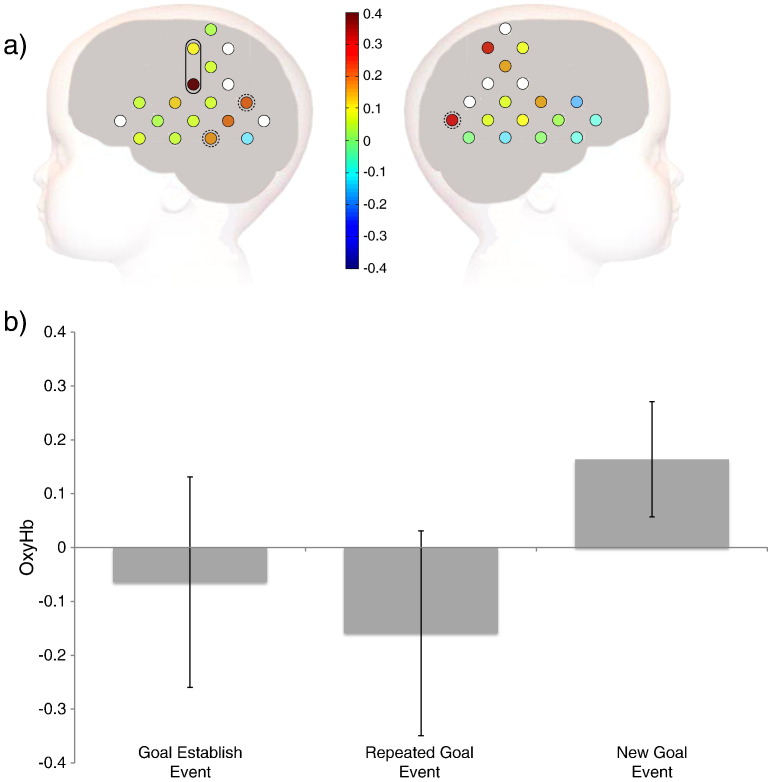
Haemodynamic responses (oxyHb) measured in our 9-month-old sample. a) Schematic head showing the difference in parameter estimates between New Goal trials and Repeated Goal trials at each channel. Positivity indicates a greater activation for New than Repeated Goal trials. The two statistically significant contiguous channels (8 and 9) are indicated with a black ring around. The three additional channels which were significant in isolation (10, 18 and 38) are marked with a dashed line around. Corresponding channel numbers are indicated on Fig. 2. Channels which were excluded from analysis due to poor data quality are shown in white. b) Bar plot of activity averaged over channels 8 and 9 for the goal-establishing event, the Repeated Goal event and the New Goal event.

In addition, the videos of the infants' behaviour during data recording were blindly coded for looking-time, to ensure infants were equally attending to all types of presented trials. There were no differences in the time infants spent looking at the stimuli between New Goal and Repeated Goal trials (*t* = .569, *p* = .577), nor between New Path and Repeated Path trials (*t* = .227, *p* = .823). We calculated the proportion of data per infant that was removed on the basis of inattention and/or movement artefact and excluded any infants for whom more than 30% of their data was excluded (n = 5). We also excluded from analysis any channels that did not yield clean data in at least 70% of infants. This resulted in the exclusion of 8 channels (marked as white circles on Fig. 3).

The preprocessed data was then converted from raw signals to oxygenated-Hb, deoxygenated-Hb, and total-Hb concentrations using the modified Beer–Lamberts law as implemented in the SPM-NIRS toolbox. For each infant, a design matrix was built which modelled six cognitive conditions. First, we created six regressors, each with the same length as the recorded data and a value of 0 at every timepoint. The first regressor modelled the two goal-establishing events, and values in this vector were set to 1 at each timepoint when a goal-establish event was on the screen. This gives a series of ‘boxcars’ each with 15 second duration. The second regressor modelled the New Goal events, with values of 1 whenever a New Goal video was on the screen, giving boxcars with 7.5 s duration. In the same way, the third regressor modelled the Repeated Goal events giving boxcars with 7.5 s duration. The fourth regressor modelled baseline periods between trials, giving boxcars with an 8 second duration. The fifth regressor modelled ‘invalid trials’, which were defined as trials where the baby did not attend for 50% of at least one of the goal-establishing events and/or 50% of the test event. In such trials, the relevant boxcar was removed from the ‘New Goal’ or ‘Repeated Goal’ regressor and placed instead in the ‘invalid trial’ regressor. The sixth regressor marked any time when the infant was not attending to the video with a value of 1, giving a series of boxcars of variable length.

These six regressors were then convolved with the standard haemodynamic response function and its temporal and spatial derivatives to make the design matrix (Friston et al., 2011). This is a standard procedure which turns the model of what events were presented to the baby into a model of what haemodynamic response should be expected in the brain, taking into account delays in BOLD response. Thus, the final design matrix had 18 columns (6 conditions, with a HRF, temporal derivative and spatial derivative for each) modelling the goal-establish; New Goal; Repeated Goal; baseline; invalid trials and non-attending time over the complete data recording session for each infant.

For each of the 3 Hb measures, this design matrix was fit to the data using the general linear model as implemented in the SPM-NIRS toolbox. Beta parameters were obtained for each infant for each of the six regressors for the HRF and the temporal and spatial derivatives. The beta parameters were combined by calculating the length of the diagonal of a cuboid where the length of each side is given by one of the three beta parameters (Calhoun et al., 2004). This allows us to consider effects arising with a typical timecourse (standard HRF) but also those with a slightly advanced or delayed timecourse (temporal derivative) or an atypical duration (dispersion derivative) in a single model. The combined betas were used to calculated a contrast for the New Goal > Repeated Goal for each infant. This contrast was then submitted to statistical tests and plotted in the figures. As in previous infant NIRS studies, our analysis is based on changes in oxyHb. Whilst studies with adults typically find that increases in oxyHb are accompanied by a decrease in deoxyHb, studies with infants typically do not find any statistically significant deoxyHb changes (Grossmann et al., 2012; Lloyd-Fox et al., 2010).

To ensure statistical reliability, we considered that activation at a single channel would be meaningful only if there is also significant activation at a spatially contiguous channel (Lloyd-Fox et al., 2011). Monte-Carlo simulations using this criterion on our dataset revealed that a per-channel threshold of *p* < 0.0292 gives a whole-array threshold of *p* < 0.05 for finding two adjacent channels activated by chance. Therefore, we only considered the effects present at *p* < 0.0292 in two adjacent channels to be significant results.

## Results

We conducted *t*-tests on the HRF contrast for the effect of Goal (Novel Goal > Repeated Goal) at each channel. Several channels exhibited significant RS (greater activation in response to viewing the New Goal events than the Repeated Goal events) for the identity of the object goal approached by the red cone (see Table 1 and Fig. 3), but only two of these channels were contiguously located and met the *p* < 0.0292 channel threshold. Channels 8 and 9, found over the left anterior part of the parietal cortex (including intraparietal sulcus), were significantly more active for New Goal than for Repeated Goal trials. A one-sampled *t*-test on data averaged over channels 8 and 9 revealed a significantly greater activation in response to viewing New Goal than Repeated Goal trials [*t* (17) = 2.41, *p* = .028]. We also conducted an equivalent analysis of RS for movement path (New Path > Repeated Path) but these analyses did not yield any significant findings.

## Discussion

In the current study, we sought to identify regions of the infant brain that are involved in the representation of action goals. Employing a modified paired RS paradigm, we demonstrate that observation of an agent repeatedly performing an action on the same goal object results in suppression of the BOLD response in a region of the infant brain that is approximately located over the left anterior region of the parietal cortex, whilst observation of the agent approaching a new goal results in a release from suppression in this region. As we controlled for the trajectory of the action (whether the red cone approached the goal object from the same side as in the goal-establishing event, or from a new side of the display), we can isolate the agent's goal as the factor that modulated brain activity in this region. Thus, our results suggest that the left anterior parietal region is involved in goal representation in the infant brain.

Notwithstanding the limitations of cortical localization estimation based on the methods used here, our results bear a strong resemblance to those previously reported in adults. Specifically, the response pattern and location of activation found in our study is similar to that found in previous work on the representation of immediate goals in adults (Hamilton and Grafton, 2006; Hamilton and Grafton, 2007b; Ramsey and Hamilton, 2010). When adults observe a human hand reaching for a previously chosen goal object versus a novel goal object, the left anterior intraparietal sulcus (aIPS) exhibits greater activation for the novel goal event than the repeated goal event (Hamilton and Grafton, 2006). Thus, both 9-month-old infants and adults appear to recruit similar brain regions when representing others' goals. Whilst our study with infants used animated shapes rather than human hands, data from adults show that the left aIPS is engaged both when the agent is a human hand and when it is an animated shape (Ramsey and Hamilton, 2010), suggesting that both may be processed in a similar manner. One further limitation of our interpretation is that we were unable to analyze data in channel 27, the right hemisphere equivalent of channel 8 which showed the strongest effects on the left. Whilst adult data suggests that the immediate goal of an action (e.g. the object which the agent is approaching) activates the left aIPS, a role for the right aIPS in goal understanding has also been identified. Specifically, when adults observe repeated action outcomes (e.g. opening or closing a box), the right aIPS exhibits RS (Hamilton and Grafton, 2007a). Thus, whilst the action goals that infants are presented with in our study mirror those that have resulted specifically in left aIPS activation in adults, an absence of data at channel 27 means that we cannot argue conclusively that our effect is left lateralized.

Finally, whilst we used the two-contiguous-channel criterion (Lloyd-Fox et al., 2011) for accepting activation as statistically important, a number of isolated channels did reach statistical significance. Currently, there is no established consensus concerning the most appropriate way to analyse infant *f*NIRS data or to correct for multiple comparisons (Minagawa-Kawai et al., 2008), and different authors have used different practices (e.g. Minagawa-Kawai et al., 2011; Wilcox et al., 2010). Thus, it is possible that a different, or less conservative criteria, would have highlighted effects in single channels as statistically important. Furthermore, it is possible that the small and inevitable variation in channel placement depending on infants' head circumferences introduced noise in to the data which may have weakened activation in some channels that might have otherwise have formed a contiguous pair. Fig. 3a indicates a line of 3 channels (10, 14 and 18) for which there is more activity for New Goal than Repeated Goal trials. However, whilst channels 10 and 18 exhibit significant effects at the single channel level, the effect at channel 14 does not reach statistical significance and so, under our criterion, the effects at channels 10 and 18 are not interpreted as statistically important. Similarly, channel 38 located over right hemisphere exhibits greater activation for New than Repeated Goal trials. These temporal channels are likely to lie over the left and right posterior part of the superior temporal sulcus (pSTS) which has been implicated in various aspects of social processing, including the processing of information relevant to others' goals (Pelphrey et al., 2004) and is considered part of the Action Observation Network (AON) (Kilner, 2011). However, previous RS studies comparing activation to novel and repeated goals have not identified the STS as being involved in encoding goals in adults (Hamilton and Grafton, 2006; 2007a; Ramsey and Hamilton, 2010). Nevertheless, one theoretical position holds that the focus of activation narrows over development as areas of the cortex become increasingly specialized for processing particular types of stimuli (Johnson et al., 2009) and recent data support this position in the domain of face processing (Cohen Kadosh et al., 2010). Thus, whilst our criterion for interpreting channel activation has highlighted the left anterior parietal region as important for goal processing in infancy as it is in adults, further studies are needed to establish whether other cortical regions might also be involved early in development.

### Implications of results

Whilst numerous studies have demonstrated that human infants structure observed movements in terms of goals from an early age, there is much debate surrounding the mechanisms involved in early goal understanding. One view is that early goal understanding is based on first person experience performing goal-directed actions (Woodward, 2009). This is based on a growing number of studies demonstrating a relationship between infants' action competence and their ability to interpret actions as goal-directed (Cannon et al., 2011; Falck-Ytter et al., 2006; Kanakogi and Itakura, 2011; Sommerville et al., 2005; Woodward, 1998). This dependence on self-experience has been interpreted as evidence that the mechanism that underlies early goal understanding is one that maps observed movements on to a pre-existing motor representation of that action in the observer (Casile et al., 2011; Falck-Ytter et al., 2006; Kanakogi and Itakura, 2011). However, many behavioural studies show that infants can represent the goals of non-human agents whose movements would not be possible to map on to any existing motor representation (Biro and Leslie, 2007; Csibra, 2008; Gergely et al., 1995; Hernik and Southgate, 2012; Luo and Baillargeon, 2005; Southgate and Csibra, 2009). The current results provide further evidence that infants can encode the goals of non-executable actions. Furthermore, they suggest that goal representation in the anterior parietal region is not dependent on matching observed actions to a corresponding motor representation.

An alternative view is that infants are sensitive to various cues which indicate that an action is goal-directed. These proposed cues include repeated action on the same object, movement directed towards one object over another, and movement which is efficiently related to an outcome. However, there has been debate over the importance of these different cues for action interpretation (Hernik and Southgate, 2012), whether these cues lead to the same goal representation (Biro et al., 2011) and it has often been assumed that some of these cues are important despite an absence of evidence (Luo and Beck, 2009; Premack and Premack, 1994). The finding that the anterior parietal region is involved in goal representation in infants provides an opportunity to elucidate the nature of infant goal representation. Future studies can test if this anterior parietal region, which our data demonstrates is responsive to the identity of the goal in 9-month-old infants, is equally responsive to different combinations of cues to the goal. For example, it is commonly held that repeated action on an object is a sufficient cue for goal attribution (Premack and Premack, 1994), yet repeated action on an isolated object does not seem to lead to an enduring goal representation once that object is paired with a new object, because according to these authors, the infant cannot continue to assume that that object is the agent's goal when they have no information about the agent's disposition towards this novel object (Luo and Baillargeon, 2005, 2007). This view has implications for the role of goal attribution in action prediction. If goal attributions do not endure in the face of novel potential targets, then it implies that goal attributions are not a good foundation on which to predict others' behaviour since it is very likely that there will frequently be new potential targets for which the infant has no information concerning the agent's disposition (Hernik and Southgate, 2012). Currently we do not know whether infants are generating a goal attribution when they observe an agent acting on a solitary target, but fNIRS may provide a means of elucidating these issues. For example, would the anterior parietal cortex exhibit RS if the red cone repeatedly approached a solitary blue cube, suggesting that the blue cube is indeed represented as the agent's goal? Or, would there be an absence of RS in this case, suggesting that such a scenario presents insufficient cues for goal attribution?

Finally, our demonstration that infants recruit similar cortical regions during the observation of an agent pursuing a goal adds credence to the interpretation of behavioural studies. Whilst many behavioural studies have concluded that infants do interpret others' actions as goal-directed, other authors have argued that such looking-time data only provide evidence for infants abilities to form statistical associations during the course of an experiment (Sirois and Jackson, 2007). Our data suggest that the infant brain not only shows a parallel pattern of repetition suppression to the adult brain, but also shows this pattern in equivalent brain regions. This suggests that 9-month-old infants are beginning to use adult-like parietal brain networks to encode the events they see in terms of action goals.

## Conclusions

This study demonstrates RS for immediate goals in the left anterior parietal cortex in 9-month-old infants, a finding which mirrors that reported when adults view similar stimuli (Hamilton and Grafton, 2006; Hamilton and Grafton, 2007a; Ramsey and Hamilton, 2010). This suggests that the left anterior parietal cortex is already specialized for goal representation in the first year of life and provides additional support for the interpretation of behavioural studies. Moreover, the fact that a region of the infant cortex appears specialized for goal representation provides an invaluable tool by which to investigate the cues and mechanisms by which infants are able to make sense of others' actions.

## Figures and Tables

**Table 1:** Channels that exhibited significant effects (*p* < .05). 5 channels exhibited a greater response to New Goal than Repeated Goal trials. No channels exhibited a statistically significant reverse effect. The spatially contiguous channels on which results are based are channels 8 and 9.

Channel	*t* value	*p* value
8	2.57	0.020
9	2.49	0.025
10	2.59	0.020
18	3.19	0.007
38	3.37	0.006
